# Do women attending antenatal clinics who use only intermittent preventive treatment (IPTp) have higher *Plasmodium falciparum* prevalence compared to those who used both IPTp and slept under insecticide-treated nets?

**DOI:** 10.1186/s12936-025-05532-1

**Published:** 2025-08-28

**Authors:** Jesuspower Chikere Madukwe, Ekenedirichukwu Blasingame Ahaneku, Okechukwu Shedrach Onukafor, Sabastine Edokpayi, Precious Chidumga Madukwe, Ngozi Uloma Enwereji, Ellen M. Santos

**Affiliations:** 1 Department of Applied Health, Southern Illinois University Edwardsville, Edwardsville, USA; 2 Federal Medical Center, Owerri, Nigeria; 3 Abia State Specialist Hospital and Diagnostic Center, Umuahia, Nigeria; 4 Temple Clinic, Asaba, Nigeria; 5 Military Pensions Board, Medical Reception Station, Abuja, Nigeria

**Keywords:** Malaria, Nigeria, *Plasmodium falciparum*, Antenatal care, Intermittent preventive treatment in pregnancy (IPTp), Insecticide-treated nets (ITNs)

## Abstract

**Background:**

As global malaria incidence continues to increase across sub-Saharan Africa, tightening usage of existing prevention strategies is crucial for protection of vulnerable populations. Nigeria alone accounted for more than a quarter of the 247 million cases in 2021. During pregnancy, Intermittent Preventive Treatment (IPTp) and use of Insecticide-treated nets (ITNs) are recommended for malaria prevention.

**Methods:**

This study sought to determine whether the combined use of IPTp and ITNs offered better protection from *Plasmodium falciparum* infection compared to a single strategy alone. A cross-sectional study of 143 pregnant women was carried out among three hospitals across the southern and southeastern regions of Nigeria. IPTp and ITN usage was measured, and *P. falciparum* prevalence was determined by rapid diagnostic testing.

**Results:**

*Plasmodium falciparum* was detected in 65 (45%) of participants; prevalence was higher among those who used only Intermittent Preventive Treatment (IPTp) (61.2%) compared to those who used both IPTp and slept under ITNs (17.0%). Logistic regression analyses adjusted for gestational week, income, and education revealed that those using IPTp alone were not significantly protected from infection, while those using IPTp and ITNs had 86% reduced odds of infection. This reduction in odds of infection appears to be driven largely by ITN.

**Conclusions:**

The results affirm the vital role of ITN usage, both independently and in conjunction with IPT, in mitigating the risk of malaria during pregnancy. Efforts should focus on addressing access barriers, enhancing ITN dissemination through ANC services, and prioritizing educational campaigns highlighting the benefits of combined malaria prevention strategies.

## Background

Malaria incidence has increased since 2016 following significant reductions between 2000 and 2015. In 2021 there were nearly 247 million cases globally; 26.6% of these cases occurred in Nigeria alone [1]. In 2021 32% (13.3 million) pregnancies were exposed to *Plasmodium falciparum* infection in the World Health Organization (WHO) African Region. West Africa experienced the highest prevalence of *P. falciparum* exposure in pregnancy, with 40.7% infections among 16 million pregnant women [1].

Malaria during pregnancy increases risk of maternal and fetal mortality, maternal anaemia, and low birthweight [1]. As a high-risk population, malaria prevention strategies are often targeted to pregnant women and young children. Updated WHO recommendations for malaria prevention among pregnant women in endemic areas include at least three doses of intermittent preventive treatment (IPTp) with sulfadoxine-pyrimethamine at antenatal visits beginning early in the second trimester of gestation [2]. There is substantial evidence that preventing malaria during pregnancy reduces poor maternal and child health outcomes. A meta-analysis of 32 national cross-sectional studies in across Africa demonstrated that neonates of pregnant women exposed to full malaria prevention with IPTp or insecticide-treated nets (ITNs) were 0.82 times as likely as those with no prevention to experience neonatal mortality [3]. Exposure to prevention was also associated with reduced odds of low birthweight [3]. Despite strong evidence of prevention benefits, there remain multiple barriers to attaining the full three recommended IPTp doses included access to and costs of antenatal care, knowledge of IPTp, drug supply chain challenges, and drug resistance [4–8].

Sleeping under ITNs is also recommended for pregnant women in malaria endemic areas, and the known benefits of decreased low birth weight and miscarriage/stillbirth are well documented [3, 9]. Additionally, ITN use during pregnancy is known to decrease the risk of neonatal mortality [3]. Despite the known effectiveness of IPTp and of ITNs for preventing malaria, it is unclear whether combining the two strategies leads to additional protection from malaria among pregnant women. The objective of this cross-sectional study was to measure whether pregnant women who used IPTp alone had higher *P. falciparum* prevalence compared to those who used both IPTp and ITNs during pregnancy.

In southeast Nigeria, the uptake of ITNs and IPTp is suboptimal. A study in the region indicated that while awareness of ITNs exists, and actual usage is low [10]. IPTp uptake in the region varies by socioeconomic status with the wealthier women more likely to receive adequate treatment [11].

## Methods

### Study sites and study population

A cross-sectional study was carried out among three hospitals across southeast and southern regions of Nigeria including Owerri, Umuahia and Asaba. These cities are the capitals of Imo, Abia, and Delta states, respectively, and are major urban areas. The study region lies within the tropical rainforest zone, a holoendemic region that experiences the highest entomological inoculation rates of the various climatic zones within the country [12].

Pregnant women between ages 18–49 years presenting for antenatal care (ANC) or delivery appointments at the labour ward of the study hospitals were selected and approached for inclusion. Those who consented were included, and those who were presenting for emergencies were excluded from the study. The women were approached based on availability of patients and clients for antenatal care or delivery appointments, with patients presenting as emergencies excluded (Table 1).

**Table 1:** Descriptive characteristics of the study participants

Patient characteristics *N* = 143	RDT^#^ Result			
	Negative (*n* = 78)		Positive (*n* = 65)	
	Mean	SD	Mean	SD
Age (Years)	29	5	29	5
Gestational Week	22	7	24	8
Monthly Household Income (Naira)	292,468	272,570	194,203	152,110
Number of Antenatal Visits	4	2	3	2
No. of Bed Nets in Household	1	1	1	1
No. of people who sleep in the home nightly	3	1	3	2
No. of people who sleep under ITNs in household	2	1	1	1

		Freq	%	Freq	%
Ability to Read and Write	Easily	76	97.4%	57	87.7%
	With Difficulty	2	2.6%	8	12.3%
Highest Class Completed	Complete Primary	0	0.0%	4	6.2%
	Incomplete Secondary	2	2.6%	7	10.8%
	> Complete Secondary	76	97.4%	54	83.1%
Skipped Meals so others could eat	No	70	89.7%	55	84.6%
	Yes	8	10.3%	10	15.4%
Knowledge of IPT	No	10	12.8%	15	23.1%
	Yes	68	87.2%	50	76.9%
IPT Adherence	No	14	46.7%	16	53.3%
	Yes	64	56.6%	49	43.4%
Frequency of ITN use in Pregnancy (nights per week)	Never (0)	25	32.1%	47	72.3%
	Rarely (1)	6	7.7%	8	12.3%
	Sometimes (2–3)	6	7.75%	7	10.8%
	Several (4–6)	23	29.5%	2	3.1%
	Every Night (7)	18	23.1%	1	1.5%
Effective ITN Use*	No	35	44.9%	56	86.2%
	Yes	43	55.1%	9	13.8%
Utilize both IPTp and ITN in combination	No	39	40.6%	57	59.4%
	Yes	39	83.0%	8	17.0%
Utilize only IPT	No	51	68.0%	24	32.0%
	Yes	26	38.8%	41	61.2%
History of symptomatic malaria in current pregnancy	No	57	73.1%	19	29.2%
	Unsure (Not Tested)	4	5.1%	1	1.5%
	Yes	17	21.8%	45	69.2%

^*^Slept under an ITN the previous night; ^#^Malaria Rapid Diagnostic Test; Freq = Frequency; SD = Standard Deviation

### Data collection

A multi-item pretested interviewer-administered questionnaire adapted from available literature was used to collect data from the study participants on socio-economic status, ANC attendance and IPTp usage, ITN usage, and nutrition. IPTp adherence was defined as receiving the recommended doses as appropriate for current gestational age. ITN usage was defined as those who slept under an ITN the previous night. Pretesting was conducted in two stages. First, a preliminary review of the instrument was conducted by the clinical collaborators and secondly, a pre-test was conducted with a small sample of patients presenting to the antenatal clinic. The questionnaire was administered to the study participants using face-to-face interviews conducted by the trained research assistants. Each questionnaire took about 30 min to administer, and data collection took place over six months between February and July 2023. Additional data was collected from the client or patient medical charts. Blood samples were collected to assess *P. falciparum* prevalence. Blood samples were collected from each participant under sterile conditions by skin prick. Sample collection was performed at the same time as the routine pre-partum sample collection for blood studies. Samples were labelled with a unique identification number to ensure participant anonymity and avoid errors in sample handling. Blood samples were analysed for malaria infection using the malaria Rapid Diagnostic Test (RDT). The RDT is a lateral flow immunoassay that detects the presence of *P. falciparum* histidine-rich protein-2 and/or lactate dehydrogenase in human blood. To maintain the consistency of results, a single brand of RDT, “Global” was used throughout the study, at all research sites.

### Data management and ethical approval

Research assistants were trained medical personnel who also served as collaborators. The research assistants were fluent in English and Igbo languages (to enable proper interpretation of the study instrument if needed). They received online training on the study instrument after completing the required ethical training for Human Subjects research. They then took part in the pretesting of the questionnaire to assess the success of and to consolidate on the training. There were online check-in meetings of the data collection team at the end of each data collection week to share experiences, submit completed forms, and ensure field problems were solved.

Ethical approval was obtained from the Federal Medical Centre, Owerri Research Ethics Committee, as well as those of Abia State Specialist Hospital and Diagnostic Centre, and Temple Clinic. The Institutional Review Board of the Southern Illinois University Edwardsville, Illinois, USA also granted ethical approval under approval number #1761. Informed consent was obtained from each participant before administering the questionnaire and collecting finger prick blood samples. Participants were informed of the purpose of the study, their rights, and the confidentiality of the information provided. They were also free to withdraw from the study at any point. The study was conducted in accordance with the ethical principles outlined in the Declaration of Helsinki. To protect patient privacy, a limited dataset containing the deidentified minimal data necessary for interpreting and replicating findings is available upon reasonable request to the corresponding author.

### Data analysis

Sample size was estimated using epidemiological sample size formulas. To detect a 20% difference in malaria prevalence between women who used preventive strategies during pregnancy and those who did not with 80% power, a sample of 194 participants was required. Out of the anticipated 280 patients presenting for ANC or labour appointments during the study period, a response rate of 70% was anticipated.

Data analysis was performed using SPSS version 23 (IBM SPSS, Chicago, IL, USA). The descriptive statistics of the study population were calculated, including mean and standard deviation for continuous variables and frequency and percentage for categorical variables. Crude and adjusted multivariate logistic regression models were used to measure the association between IPTp alone and ITN use alone with *P. falciparum* prevalence. Crude and adjusted univariate logistic regression models were used to measure the association between adherence to both IPTp & ITN use with *P. falciparum* prevalence. Adjusted models controlled for gestational week, income, and education. Assumptions for logistic regression were tested and confirmed, including independent observations and absence of multicollinearity. Odds ratios and 95% confidence intervals were reported.

## Results

A total of 159 women were recruited across three facilities in the region; 43.5% from Abia Specialist Hospital; 29.8% from Temple Clinic and 26.7% from Federal Medical Centre, Owerri. A total of 143 pregnant women were included in the study after data cleaning. The mean age of the participants was 29 years (SD = 5). Out of the 143 participants, 78 (54.5%) tested negative for *P. falciparum* using the malaria RDT, while 65 (45.5%) tested positive. The *P. falciparum* prevalence rate was higher among pregnant women who used only IPTp (61.2%) compared to those who used both IPTp and slept under ITNs (17.0%).

The average gestational age was 22 weeks (SD = 7) for the RDT negative cohort and 24 weeks (SD = 8) for the RDT positive cohort, representing the varying stages of pregnancy within the entire cohort. It is imperative to note that eligible subjects were at least 13 weeks 0 days of gestation, to account for the typical timing of the first IPTp dose.

The monthly household income, an indicator of the economic background of the participants, varied largely between participants with RDT negative results (292,468 Naira; SD = 272,570) and those with RDT positive results (194,203 Naira; SD = 152,110). Participants who had positive RDT results had a significantly lower monthly household income. Additionally, in contrast to 10.3% of participants with negative RDT results who skipped meals so others in the household could eat, the frequency of skipped meals for participants who had positive RDT results was higher at 15.4%.

The educational status of the participants revealed that 97.4% were able to read and write with ease. Furthermore, the same proportion of participants (97.4%) had completed at least a secondary level of education, alluding to a reasonable literacy level among the participants, and the potential to comprehend targeted healthcare education and instructions. Of those who tested positive on RDT, a greater proportion had no knowledge of IPTp (23.1%). Conversely, a lesser proportion of participants who tested positive on RDT complied with IPTp (43.4%).

### Association of IPTp and ITN use with *Plasmodium falciparum* prevalence

Crude logistic regression analyses showed that the use of ITNs was significantly associated with lower odds of *P. falciparum* infection (OR = 0.13, 95% CI: 0.06, 0.30) (Table 2). Similarly, after controlling for gestational week, income, and education, the adjusted logistic regression model reaffirmed the significant association between ITN use and lower *P. falciparum* prevalence (OR = 0.15, 95% CI: 0.06, 0.38) (Table 3). In contrast, IPTp alone did not show a significant association with *P. falciparum* prevalence in both the crude and adjusted logistic regression models.

**Table 2:** Crude logistic regression results for the association between IPTp, ITNs, and both with RDT result

Multivariate Model	OR	95% CI
IPTp	1.05	0.44, 2.54
ITN Use	0.13	0.06, 0.30
Univariate Model		
IPTp & ITN Use	0.14	0.06, 0.33

OR = Odds Ratio; CI = Confidence Interval

**Table 3:** Adjusted logistic regression results for the association between IPTp, ITNs, and both with RDT result

Multivariate Model	OR	95% CI
IPTp	1.14	0.41, 3.14
ITN Use	0.15	0.06, 0.38
Univariate Model		
IPTp & ITN Use	0.16	0.07, 0.40

OR = Odds Ratio; CI = Confidence Interval. Models adjusted for gestational week, income, and education

In addition, adherence to both IPTp and ITN use was associated with significantly lower odds of *P. falciparum* infection (OR = 0.14, 95% CI: 0.06, 0.33) in the crude logistic regression model. This association remained significant in the adjusted logistic regression model (OR 0.16 95% CI: 0.07, 0.40) (Table 3).

The results of the study showed that pregnant women who used IPTp alone had a higher *P. falciparum* prevalence as compared to those who adopted both IPTp and ITNs. Notably, the odds of having malaria were significantly lower among pregnant women who used ITNs, either alone or in combination with IPTp.

## Discussion

Malaria in pregnancy continues to pose a significant public health challenge to Nigeria, among other countries in sub-Saharan Africa. Efforts to bridge the gap in uptake of Antenatal care (ANC), IPTp, ITNs, and other ancillary preventive strategies have proven slow in development. With only 63% ANC coverage, 31% IPTp coverage, and 50% ITN use among pregnant women across Nigeria so far, there remains a lot to do in ensuring the vision of a combined package of prevention [13].

This study investigated whether pregnant women using both IPTp and ITNs showed a lower malaria prevalence than those using only IPTp. The findings pointed out a higher *P. falciparum* prevalence among women who used only IPTp, suggesting that ITNs used alone or combined with IPTp resulted in a lower risk of malaria in pregnancy. A similar study in Cameroon found that pregnant women using both interventions had 0.34 times the odds of prevalent *P. falciparum* infection compared to those using only one control method [14]. In the present study, the reduced odds of parasitaemia appear to be driven by ITN use rather than IPTp, while Fokam and colleagues found evidence of a more synergistic effect [14].

In work by Yusuf et al*.,* it was found that more pregnancies were protected by ITNs than IPTp [15]. This disparity in protective efficacy was attributed to the higher level of ITN uptake and utilization, relative to IPTp in Nigeria. The association between ITN usage and reduced *P. falciparum* prevalence is consistent with past studies on the effectiveness of ITNs in preventing malaria in the general population and during pregnancy [16–18]. ITNs provide a physical and chemical barrier against mosquito bites, reducing the risk of malaria at both the individual and community levels. ITNs have been noted to effectively reduce the malaria risk by mechanisms which include reducing the fecundity of host-seeking mosquitoes, killing of insecticide-susceptible mosquitoes and even insecticide resistant vectors [19]. According to the WHO, by consequently reducing the density, age, and proportion of local mosquito population, pregnant women are protected at an individual and community level.

Compared to Northern Nigeria, ITN access goals in the Southeast have developed more slowly, with the reverse being the case for IPTp [15]. This disparity in the growth of the programs may be due to variations in implementation patterns in both regions. The relative successes of IPTp implementation in the study area must be escalated and applied to ITNs. The findings of high ITN effectiveness in the present study support the need for a greater push to increase ITN access and use in the Southeast region. Nationally, ITNs are distributed through mass campaigns and routine platforms such as ANC clinics, with 79% from mass distribution campaigns and only 5% obtained in ANC [13].

In contrast, the mainstay of IPTp distribution is through ANC, typically in primary, secondary, and tertiary medical facilities [20]. According to the National Malaria Elimination Programme [NMEP] [Nigeria], National Population Commission [NPC] [Nigeria], and ICF (2022), 59% of pregnant women received at least one dose of IPTp, which is lower than our study’s IPTp uptake rate of 79%. The ANC-limited sampling of the study participants may explain this higher IPTp uptake rate. However, these statistics indicate opportunities for leveraging ANC as a central implementation point for both IPTp and ITNs [15].

In addition to adopting ITN counseling, recommendation, and distribution as a standard ANC practice, the gap in access can also be closed by upscaling distribution efforts in urban areas. Paradoxically, higher *P. falciparum* infection rates, even among pregnant women, are associated with higher income, education, and urban living [15]. This is partially attributable to the lower percentage access and usage of ITNs in urban areas (41% and 33% respectively), as opposed to rural areas (44% and 38% respectively) [15]. Based on these numbers, deliberate efforts can be made to integrate IPTp and ITNs as routine ANC offerings in urban areas while increasing access to qualified ANC services in rural areas.

The role of high-quality education cannot be understated in addressing the issue of malaria in pregnancy. The education of pregnant women on the importance of uncompromisingly adopting both IPTp and ITNs is critical to improving the bottom line of access and adherence to combined strategies against malaria in pregnancy, not to mention ensuring favourable fetal-maternal outcomes. There is also a need to train and retrain healthcare workers, including health educators, to adopt uniform care guidelines, especially in ANC. The same training can also be extended to Community Health Workers, Traditional Birth Attendants, and faith-based birth attendants, who are reported to be alternative and sometimes concurrent points of care for pregnant women in Nigeria [20].

Finally, technology has a place in advancing perinatal care and mitigating malaria during pregnancy. In the Nigerian context, well-designed and expert-informed mobile technology can be applied in the education of the pregnant population and the sustenance of malaria preventive strategies. Nigeria has seen a notable increase in mobile phone usage, with an estimated 87% penetration rate, indicating widespread accessibility of mobile technology across the population [21]. This extensive reach provides an opportunity for implementing mobile health applications and other digital tools in malaria prevention. Furthermore, a study by Oyelola reported that 80% of adults in Nigeria owned some type of mobile phone, with a growing trend in the use of smartphones especially [20]. There was also a high percentage of internet users utilizing smartphones for web access (92.4%) [20]. These high mobile usage statistics emphasize the potential impact that mobile-based interventions could have in Nigeria [22].

The far-reaching implication is that mobile health platforms could be effectively used to deliver educational content, track adherence with IPTp and ITN use, and facilitate remote healthcare services. With much of the population increasingly gaining access to mobile technology, implementing such interventions could significantly enhance malaria prevention efforts among pregnant women in Nigeria.

### Limitations

The final sample size was lower than anticipated from power calculations. A sample size of 194 participants was estimated to be necessary to detect a 20% difference in malaria prevalence between groups, and the resulting sample size was 143 participants. The smaller than expected sample size may have limited the ability to detect statistically significant differences in the resulting analyses. Future research should aim for a larger, more diverse sample to strengthen and corroborate these findings.

Additionally, the study sample included only women attending ANC appointments at tertiary facilities which may limit the generalizability to the general population of pregnant women in this region. This likely explains the findings of high reported IPTp adherence and may limit the ability to measure the synergistic effect of IPTp and ITNs.

## Conclusions

This study highlights the critical importance of combining Intermittent Preventive Treatment in pregnancy (IPTp) with Insecticide-Treated Nets (ITNs) as an effective strategy for malaria prevention among pregnant women. The findings reveal that women who used both IPTp and ITNs had a significantly lower prevalence of *P. falciparum* compared to those who relied solely on IPTp. The research distinctly affirms the vital role of ITN usage, both independently and in conjunction with IPTp, in mitigating the risk of malaria during pregnancy. These results support the recommendation for an integrated approach to malaria prevention, advocating for the simultaneous implementation of IPTp and ITNs within Antenatal Care (ANC) services.

Future efforts should focus on addressing access barriers, enhancing ITN dissemination through ANC services, and prioritizing educational campaigns highlighting the benefits of combined malaria prevention strategies. These efforts align with the study's findings, emphasizing the potential of integrated IPTp and ITN utilization to mitigate the impact of malaria on maternal and fetal health.

In summary, the study’s outcomes advocate for the integration of IPTp and ITNs within ANC programs, demonstrating promise in substantially reducing malaria prevalence among pregnant women in malaria-endemic regions. Further community-level studies are needed to assess the prevalence of malaria against the backdrop of these preventive strategies on a broader scale, especially in diverse geographical settings. Additionally, these studies should aim to capture the geopolitical variations in malaria prevalence across different regions of Nigeria, to provide a more comprehensive understanding.

## Data Availability

The datasets used and/or analysed during the current study are available from the corresponding author upon reasonable request.

## Abbreviations

IPTp: Intermittent preventive treatment in pregnancy
ITNs: Insecticide-treated nets
WHO: World Health Organization
ANC: Antenatal care
RDT: Rapid diagnostic test
NMEP: National malaria elimination programme
NPC: National Population Commission
